# Rapid Analysis of the Chemical Compositions in *Semiliquidambar cathayensis* Roots by Ultra High-Performance Liquid Chromatography and Quadrupole Time-of-Flight Tandem Mass Spectrometry

**DOI:** 10.3390/molecules24224098

**Published:** 2019-11-13

**Authors:** Li Yang, Rong-Hua Liu, Jun-Wei He

**Affiliations:** 1 Key Laboratory of Modern Preparation of TCM, Jiangxi University of Traditional Chinese Medicine, Ministry of Education, Nanchang 330004, China; yangli07971@163.com; 2 College of Pharmacy, Jiangxi University of Traditional Chinese Medicine, Nanchang 330004, China; 3 Research Center of Natural Resources of Chinese Medicinal Materials and Ethnic Medicine, Jiangxi University of Traditional Chinese Medicine, Nanchang 330004, China

**Keywords:** *Semiliquidambar cathayensis* roots, UHPLC-Q-TOF-MS/MS, chemical profiling, rapid identification, chemical compositions

## Abstract

*Semiliquidambar cathayensis* Chang was a traditional medicinal plant and used to treat rheumatism arthritis and rheumatic arthritis for centuries in China with no scientific validation, while only 15 components were reported. Thus, a rapid, efficient, and precise method based on ultra-high-performance liquid chromatography coupled with quadrupole time-of-flight tandem mass spectrometry (UHPLC-Q-TOF-MS/MS) was applied in both positive- and negative-ion modes to rapidly analysis the main chemical compositions in *S. cathayensis* for the first time. Finally, a total of 85 chemical compositions, including 35 alkaloids, 12 flavonoids, 7 terpenoids, 5 phenylpropanoids, 9 fatty acids, 7 cyclic peptides, and 10 others were identified or tentatively characterized in the roots of *S. cathayensis* based on the accurate mass within 5 ppm error. Moreover, alkaloid, flavonoid, phenylpropanoid, and cyclic peptide were reported from *S. cathayensis* for the first time. This rapid and sensitive method was highly useful to comprehend the chemical compositions and will provide scientific basis for further study on the material basis, mechanism and clinical application of *S. cathayensis* roots.

## 1. Introduction

*Semiliquidambar cathayensis* Chang, is an epiphyllum tree belonging to the Hamamelidaceae family, native only to China, and grows in Jiangxi, Guangxi, Guangdong, Hainan and Guizhou [1]. Chinese people call the roots of *S. cathayensis* as *Ban feng he* (Chinese name 半枫荷), which have long been used in traditional Chinese medicine (TCM) for the treatment of rheumatism arthritis and rheumatic arthritis [2]. Modern pharmacological experiments have demonstrated that the crude extracts and/or fractions obtained from the roots of *S. cathayensis* have the effects of analgesia, anti-inflammatory, anti-hepatitis B virus, promoting blood circulation, and removing blood stasis [3,4,5,6]. Unfortunately, only 15 chemical compositions—including 7 terpenoids, 3 steroids, 3 tannins, and 2 fatty acids—were reported from the roots of *S. cathayensis* [7,8], which was a significant barrier for further pharmacological, metabolic and pharmacokinetic studies of this medicinal plant. Moreover, due to the indeterminate relationship between pharmacological activities, chemical components, the clinical application and quality control of *S. cathayensis* roots still faced big challenges. Therefore, a rapid and sensitive method to figure out the chemical components in the roots of *S. cathayensis* was urgently needed.

Conventional separation and identification processes were time and plant material consuming [9,10,11,12,13], whereas the use of a rapid, efficient, and prescise method focused on identification chemical components was very important for TCMs. Over the past decade, UHPLC coupled with high-resolution mass spectrometry (HRMS) has become the prime tool for investigating the chemical profiling of TCMs, because of its advantages on the peak capacity, resolution, separation time, and detection sensitivity, all of which are suitable for addressing the complicated characteristics of the constituents in TCMs [14,15,16,17]. Furthermore, quadrupole time-of-flight Q-TOF-MS/MS with powerful structural characterization can provide more specific and accurate mass measurements for both precursor and fragment ions. These features can greatly facilitate prediction of elemental compositions and fragmentation pathways [18,19,20].

In this study, a rapid, sensitive, and reliable approach based on UHPLC-Q-TOF-MS/MS method was established to determine the main chemical components in the roots of *S. cathayensis* for the first time, which will provide a basis for further study in vivo of *S. cathayensis* roots and the information of potential new drug structure for treating rheumatism arthritis and rheumatic arthritis.

## 2. Results and Discussion

### 2.1. Optimization of Chromatographic Separation

**Figure 1:**
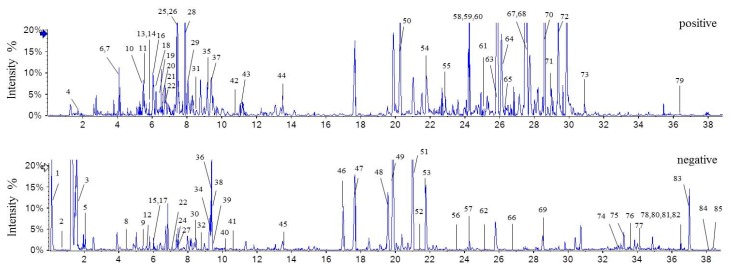
The total ion chromatograms of the *S. cathayensis* roots extract by UHPLC-Q-TOF-MS/MS in positive- and negative-ion modes.

A series of parameters, including stationary and mobile phases, flow rate, and column temperature were investigated in order to obtain optimal chromatographic separation and analytical sensitivity for multiple constituents in the roots of *S. cathayensis*. A comparative study based on chromatographic selectivity and detection sensitivity revealed that the best performance was achieved with the BEH C_18_ column as the stationary phase and acetonitrile as the organic part of the mobile phase. Since alkaloid compounds generally exhibits better mass spectrometric responses in positive ionization mode, the addition of 0.1% formic acid into the aqueous part of the mobile phase was found to be beneficial to the subsequent positive electrospray ionization (ESI^+^) analysis. In addition to the optimization of the stationary and mobile phases, control of column temperature and flow rate were also optimized to improve selectivity and resolution. Finally, column temperature of 35 °C and the flow rate of 0.3 mL/min were suitable for the separation. The total ion chromatogram (TIC) of *S. cathayensis* roots extract in positive- and negative-ion modes were shown in Figure 1. Moreover, the tandem mass spectra of the main components were available in Supplementary Materials.

### 2.2. Identification of Main Constituents in S. Cathayensis Extract

**Figure 2:**
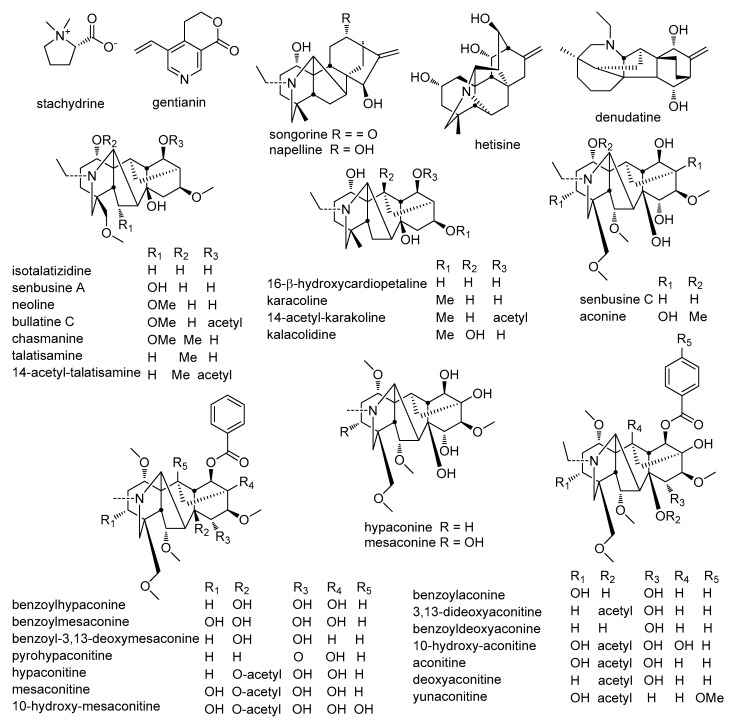
Chemical structures of alkaloids from *S. cathayensis* roots.

**Figure 3:**
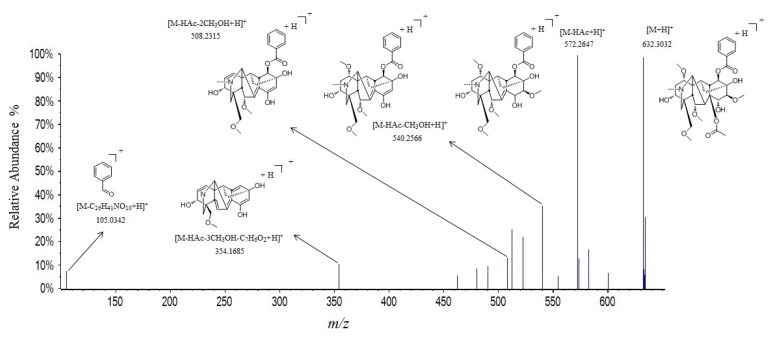
Tandem mass spectra and its fragmentation of mesaconitine in positive ion mode.

**Figure 4:**
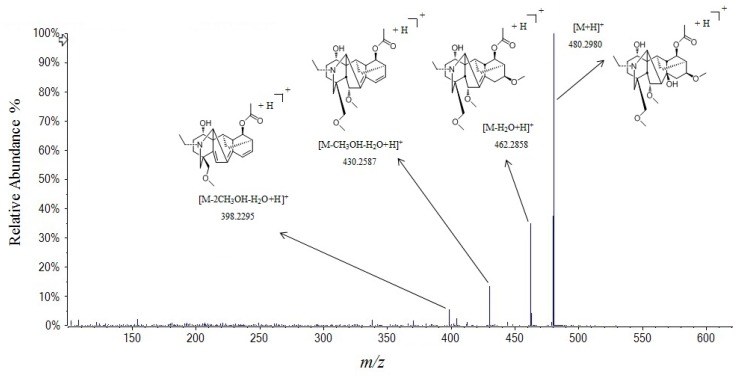
Tandem mass spectra and its fragmentation of bullatine C in positive ion mode.

**Figure 5:**
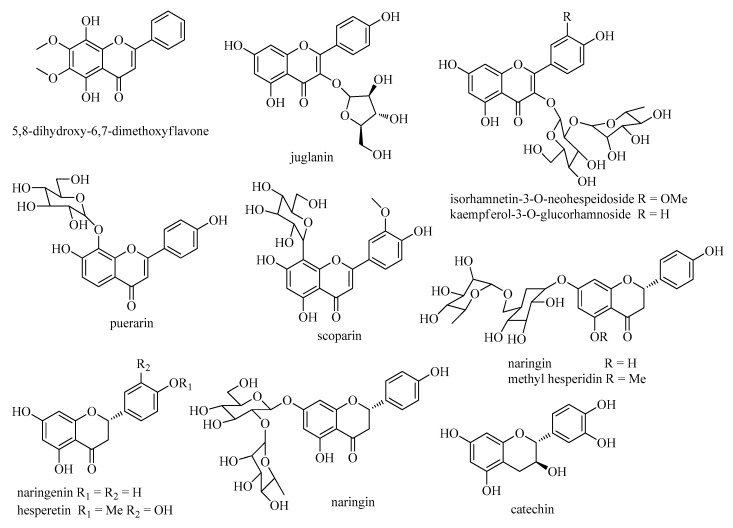
Chemical structures of flavonoids from *S. cathayensis* roots.

**Figure 6:**
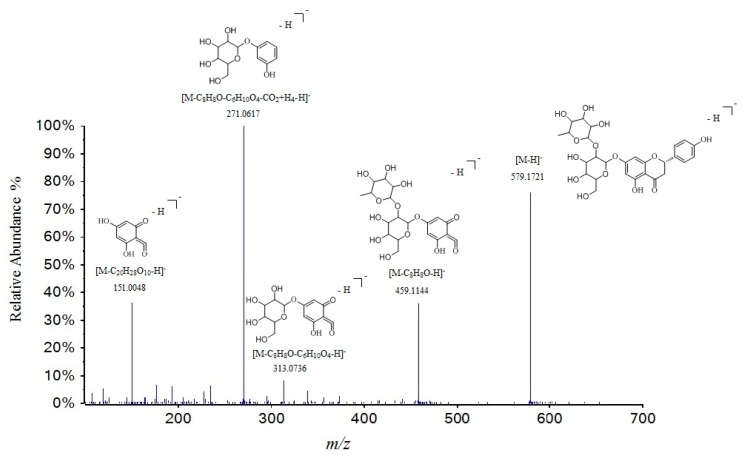
Tandem mass spectra and its fragmentation of naringin in negative ion mode.

**Figure 7:**
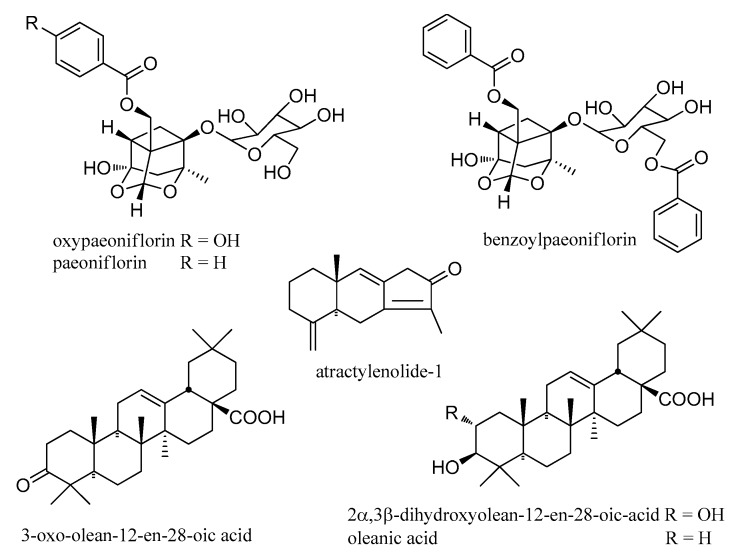
Chemical structures of terpenoids from *S. cathayensis* roots.

**Figure 8:**
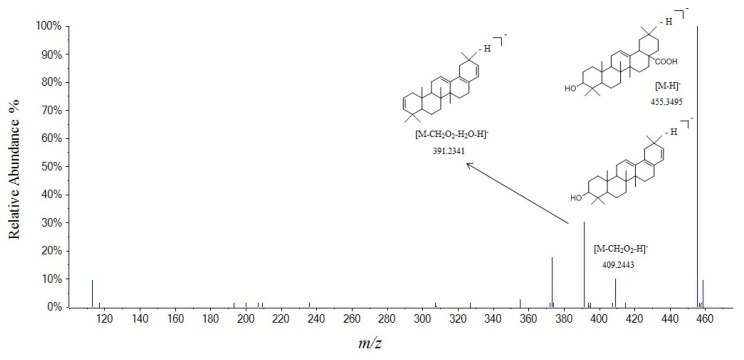
Tandem mass spectra and its fragmentation of oleanolic acid in negative ion mode.

**Figure 9:**
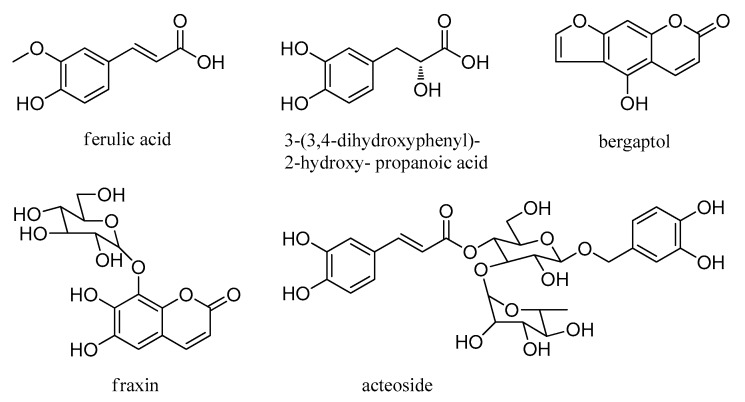
Chemical structures of phenylpropanoids from *S. cathayensis* roots.

A total of 85 chemical compositions, including 35 alkaloids, 12 flavonoids, 7 terpenoids, 5 phenylpropanoids, 9 fatty acids, 7 cyclic peptides, and 10 others were identified. The molecular formula was accurately assigned within mass error of 5 ppm. Then the fragment ions were used to further confirm the chemical structure. Information including compound name, retention time, formula, precursor ion, and fragment ions of the rest of these compositions can be found in Table 1; Table 2. The chemical structures of the main components in *S. cathayensis* roots extract are showed in Figure 2, Figure 3, Figure 4, Figure 5, Figure 6, Figure 7, Figure 8 and Figure 9. All the components were identified based on the existing literatures, which includes the database of Chinese medicine ingredients and the free chemical structure database, including ChemSpider, Massbank, and mzCloud. Furthermore, the fragmentation pathways of some compounds were proposed in order to facilitate structural identification.

#### 2.2.1. Alkaloids

A total of 35 alkaloids (Figure 2) in the roots of *S. cathayensis* extract were indentified in positive ion mode, all ones except compounds **4** and **6** were diterpenoid alkaloids, which can be classified into C_18_-, C_19_-, and C_20_- diterpenoid alkaloids according to skeleton carbons [46]. In this study, compounds **7**, **20**, **21**, **22**, and **37** were C_20_-diterpenoid alkaloids, while others were C_19_-diterpenoid alkaloids, which were belonging to aconitum alkaloids. Moreover, aconitum alkaloids include diester-diterpenoid, monoester-diterpenoid, amine-diterpenoid, and other alkaloids [25]. In tandem mass spectrum of aconitum alkaloids commonly observe the neutral losses of H_2_O, MeOH, AcOH, or BzOH. In diester-diterpenoid alkaloids, the hydroxyl groups of C8 and C14 in these compounds are combined with acetic acid and benzoic acid to form esters, respectively. The neutral loss of 28 Da corresponding to eliminate one molecule of CO or C_2_H_4_ was the feature loss. Meanwhile, the order of eliminations of benzyl, acetyl, carboxyl, ethyl, or methyl and methoxy was also investigated. In monoester-diterpenoid alkaloids, only hydroxyl group of C14 binds with benzoic acid to form esters. Then, hydroxyl at C1 position was the most active site. Unlike them, hydroxyl at C15 position could not be eliminated even at a high fragment or voltage in amine diterpenoid alkaloids [15].

Compounds **59**, **63**, **64**, **67**, **68**, **70**, **71**, and **72** were diester-diterpenoid alkaloids. Among them, **63**, **67**, and **68** showed [M + H]^+^ ion at *m*/*z* 632.3032, 616.3089 and 646.3199. They have similar fragmentation pathways, including [M + H − HAc]^+^, [M + H − HAc − CH_3_OH]^+^, [M + H − HAc − CH_3_OH − CO]^+^, [M + H − HAc − 2CH_3_OH − CO]^+^, [M + H − HAc − 2CH_3_OH]^+^, and [M + H − HAc − 3CH_3_OH − C_6_H_5_COOH]^+^, which were identified as mesaconitine, hypaconitine, and aconitine, respectively. The possible fragmentation mechanism of mesaconitine is depicted in Figure 3.

Moreover, compounds **59** and **64** gave [M + H]^+^ ion at *m*/*z* 648.3002 and 662.3168, 16 Da greater than that of mesaconitine and aconitine, respectively. As C10 position was commonly substitued by hydroxyl in aconitum alkaloids, they were presumed as 10-OH mesaconitine and 10-OH aconitine, respectively. However, compounds **70** and **72** gave [M + H]^+^ ion at *m*/*z* 630.3256 and 614.3302, 16 Da and 32 Da less than aconitine, respectively. Their tandem mass spectra were smililar to aconitine, and some fragment ions of compounds **70** and **72** were 16 Da and 32 Da less than the fragment ions of aconitine, respectively. They were reported as deoxyaconitine and 3,13-dideoxyaconitine, respectively. Compound **71** showed [M + H]^+^ ion at *m*/*z* 660.3396, 14 Da greater than that of aconitine. Moreover, as C14 position was commonly substitued by methoxybenzoyl in aconitum alkaloids, **71** was presumed as yunaconitine [24,47].

Compounds **29**, **42**, **44**, **50**, **54**, **55**, **58**, **60**, and **61** are monoester-diterpenoid alkaloids. Compound **29** gave fragment ions at *m*/*z* 420.2757 and 402.2653 were corresponding to [M + H]^+^ and [M + H − H_2_O]^+^, respectively, and it was identified as 14-acetyl-karakoline by comparing with the literature [25]. Compound **42** gave fragment ions at *m*/*z* 480.2980, 462.2858, 430.2587, and 398.2295 in the positive mode were corresponding to [M + H]^+^, [M + H − H_2_O]^+^, [M + H − H_2_O − CH_3_OH]^+^, and [M + H − H_2_O − 2CH_3_OH]^+^, respectively, and it was tentatively identified as bullatine C (Figure 4). The [M + H]^+^ ion at *m*/*z* 464.3011 of compound **44** also gave the fragment ion at *m*/*z* 432.2746 [M + H − CH_3_OH]^+^, and identified as 14-acetyl-talatisamine. Compounds **50**, **54**, **55**, and **60** were 42 Da less than mesaconitine, aconitine, hypaconitine, and deoxyaconitine, respectively. As C8 position was substitued by hydroxyl group instead of acetyl group. Therefore, they could be considered as benzoylmesaconine, benzoylaconine, benzoylhypaconine, and benzoyldeoxyaconine, respectively. Moreover, compound **58** was 32 Da less than that of benzoylmesaconine, and was identified as benzoyl-3,13-deoxymesaconine. Meanwhile, compound **61** was 60 Da less than that of hypaconitine, and was presumed as pyrohypaconitine, attributing to one molecule of acetic acid eliminated from hypaconitine.

Compounds **10**, **11**, **13**, **16**, **18**, **19**, **25**, **26**, **28**, **35**, and **43** are amine diterpenoid alkaloids. Among them, **10**, **16**, **19**, **25**, and **35** were 104 Da less than benzoylmesaconine, 14-acetyl-karakoline, benzoylaconine, benzoylhypaconine, and 14-acetyl-talatisamine; and considered as mesaconine, karacoline, aconine, hypaconine, and talatisamine, respectively, because C14 position was substitued by hydroxyl group instead of benzoyl group. Moveover, **11**, **13**, **18**, **26**, **28**, and **43** could be considered as 16-β-hydroxycardiopetaline, senbusine A, isotalatizidine, senbusine C, neoline, and chasmanine, respectively, based on their molecular weight and tandem fragment patterns.

Compounds **7**, **20**, **21**, **22** and **37** were C_20_-diterpenoid alkaloids, which gave [M + H]^+^ ions at *m*/*z* 394.2588, 358.2375, 360.2536, 330.2062 and 344.2587, respectively. Thus, they were respectively identified as kalacolidine, songorine, napelline, hetisine, and denudatine by comparing with the literatures [15,48].

The [M + H]^+^ ion of compound **4** was shown at *m*/*z* 144.1004. Its MS^2^ fragment ions at *m*/*z* 128.0647 and 102.0585 exhibited the loss of CH_4_ and continuous loss of two CH_4_, and it was presumed as stachydrine [22]. For compound **6**, the positive mode MS spectrum showed the parent ion at *m*/*z* 176.0714 [M + H]^+^, and MS^2^ spectrum showed the fragment ions at *m*/*z* 148.0763 [M + H − C_2_H_4_]^+^, 130.0660 [M + H − COOH]^+^, and 120.0820 [M + H − C_2_H_4_ − CO]^+^. Compared with literature data, compound **6** was identified as gentianin [23].

#### 2.2.2. Flavonoids

Flavonoids were a kind of basic 2-phenyl chromogenic ketones, which exist widely in nature and were important natural organic compounds. Two flavonoids (**17** and **56**) and 10 flavonoid glycosides in the roots of *S. cathayensis* extract were indentified in negative ion mode (Figure 5). For flavonoids, small molecules and radicals like CH_3_ (15 Da), H_2_O (18 Da) and CO (28 Da) were feature loss. The main MS/MS behavior of aglycones described previously was retro Diels-Alder (RDA) fragmentation pathway. RDA fragments of *m*/*z* 135 and 119 were the feature fragments in negative ion mode [20,49]. Taking compound **17** as an example, it had [M − H]^−^ ion at *m*/*z* 289.0715. It yielded fragments at *m*/*z* 205.0532, 203.0700, 187.0372, and 125.0280 by loss of 2(C_2_H_2_O), 2(C_2_H_3_O), 2(C_2_H_2_O)-H_2_O, and C_9_H_8_O_3_ moieties. It was consistent with literature and identified as catechin [35].

Moreover, the loss of a glucuronic acid (176 Da) and hexose residue (glucose 162 Da, rhamnose 146 Da) were often seen in flavonoid glycosides. Compounds **24**, **33**, **34**, **36**, **39**, **46**, **48**, **56**, **57**, **62**, and **66** were considered as puerarin, scoparin, isorhamnetin-3-*O*-neohespeidoside, kaempferol-3-*O*-glucorhamnoside, methyl hesperidin, naringin, hesperidin, 5,8-dihydroxy-6,7-dimethoxyflavone, juglanin, naringenin, and hesperetin, respectively [31,32,33,34,36]. Take compound **46** as an example (Figure 6), it had [M − H]^−^ ion at *m*/*z* 579.17156 in the spectrum. Four main fragment ions at *m*/*z* 459.1144, 271.0617, 177.0209, and 151.0048 were obviously observed. Among them, the most abundant fragment ion *m*/*z* 271.0617 was suggested by the loss of rutinose residue [M − H − 146 − 162]^−^. Fragment ions at *m*/*z* 459.1144 and 151.0048 were glycoside and aglycone by RDA. The fragment information at *m*/*z* 177.0209 was detected as aglycone without C ring. Compared to the MS spectra data and references [34,36] compound **46** was tentatively identified as naringin.

#### 2.2.3. Terpenoids

Terpenoids were a class of structures derived from methylglutaric acid (MVA) and have two or more isoprene units (C5) on the basic carbon shelf. Seven terpenoids were identified in this study, including three monoterpenes (**14**, **31**, and **65**), one sesquiterpene (**73**) and three triterpenes (**76**, **79**, and **81**) (Figure 7). Monoterpenes usually detected the neutral losses of a benzoic acid at *m*/*z* 121 or glucosyl group at *m*/*z* 165, and aglycone ions at *m*/*z* 195 or 197, or their fragmentations of losing H_2_O and CO [15]. In that case, fragmentation behaviors showed that compound **14**, **31**, and **65** were oxypaeoniflorin, paeoniflorin, and benzoylpaeoniflorin, respectively [26,27].

Compound **73** had [M + H]^+^ ion at *m*/*z* 231.13795, and its fragments were at *m*/*z* 163.0778 [M + H − C_5_H_8_]^+^, 155.0848 [M + H − HCOOH − C_2_H_6_]^+^, 143.0931 [M + H − HCOOH − C_3_H_6_]^+^, and 105.0712 [M + H − HCOOH − C_3_H_6_ − C_4_H_2_]^+^. Its fragmentation process was the same as the literature [28] and identified as atractylenolide-1.

It was reported that triterpenes (**76**, **79**, and **81**) had similar tandem fragment patterns [29,30]. Taking compound **81** as an example, it had [M − H]^−^ ion at *m*/*z* 455.3495, and its fragments were at *m*/*z* 409.2443 [M − H − CH_2_O_2_]^−^ and 391.2341 [M − H − CH_2_O_2_ − H_2_O]^−^. Its fragmentation process was the same as the literature [29,30], and identified as oleanolic acid (Figure 8).

#### 2.2.4. Phenylpropanoids

Phenylpropanoids were structures containing one or more C_6_-C_3_ units, which were widely distributed in medicinal plants. A total of five phenylpropanoids in the roots of *S. cathayensis* extract were indentified in negative ion mode (Figure 9). Compounds **15**, **22**, **32**, **38**, and **52** were considered as ferulic acid, fraxin, 3-(3,4-dihydroxyphenyl)-2-hydroxy-propanoic acid, acteoside, and bergaptol, respectively [31,33,37,38,39].

Taking compound **38** as an example, it had [M − H]^−^ ion at *m*/*z* 623.0455, and its fragments were at *m*/*z* 461.1451 [M − H − C_9_H_6_O_3_]^–^ and 315.1067 [M −H − C_9_H_6_O_3_ − C_6_H_10_O_4_]^−^. Its fragmentation process was the same as the literature [31] and identified as acteoside.

#### 2.2.5. Fatty Acids

Fatty acids found in medicinal plants vary in length chains from 12 to 22 carbon atoms, of which 16–20 carbon atoms are the most common fatty acids in nature. It was reported that these compositions have a wide range of biological activities, including stabilizing cell membranes, maintaining the balance of cytokines and lipoproteins, and fighting cardiovascular diseases. In this study, nine fatty acids with long aliphatic hydrocarbon chains and a carboxyl group at one end (compounds **5**, **69**, **74**, **77**, **78**, **80**, **83**, **84**, and **85**) were identified based on the existing literatures [37,45,46,50], and in the relevant databases, such as ChemSpider, Massbank, and mzCloud.

#### 2.2.6. Cyclic Peptides

Compounds **27**, **40**, **45**, **47**, **49**, **51**, and **53** had similar fragmentation behaviors, and showed [M + HCOO]^−^ ions at *m*/*z* 384.2488, 497.3328, 610.4168, 723.5020, 836.5862, 949.6704, and 1062.7547, respectively. According to reference mass spectra and fragmentation spectra reported in the literatures [46,51,52], a total of seven cyclic peptides were identified with 3–9 leucyl (or isoleucyl) groups in the roots of *S. cathayensis*.

#### 2.2.7. Others

Other 10 compounds **1**–**3**, **8**, **9**, **12**, **30**, **41**, **75**, and **82** were considered as glucogallin, gallic acid, sucrose, piscidic acid, vanillin, protocatechuic acid pentoside, vanillic acid, paeonol, dibutyl phthalate, and dimethisterone, respectively [32,37,40,41,42,43,44,45,46].

## 3. Experimental Section

### 3.1. Chemicals and Reagents

Acetonitrile and formic acid (LC-MS grade) were purchased from Fisher Scientific (Pittsburgh, PA, USA). Deionized water was purified by a Milli-Q ultrapure water system (Merck Millipore, Milford, MA, USA). All other regents used of at least analytical grade.

### 3.2. Plant Material and Extraction

The roots of *S. cathayensis* were collected in the town of Longsheng, Guilin City, Guangxi, China, in October 2016. A botanical voucher specimen of this plant (no. SC20161022) was deposited at authors’ laboratory and was identified by one of the authors Ronghua Liu. 1.0 g aliquots of the roots of *S. cathayensis* powders were weighed and transferred into a 100-mL conical flask. 50 mL of 50% aqueous ethanol solution was added, and then extracted with a reflux twice for 60 min. Then, the fluid was filtrated and concentrated under reduced pressure in a rotary evaporator. Subsequently, the concentrated extract was lyophilized and dissolved in ACN. The solution was filtered through a 0.22-μm PTFE membrane before submitting for instrumental analysis.

### 3.3. UHPLC-Q-TOF-MS/MS

The UHPLC analysis were carried out on a Shimadzu System (Kyoto, Japan), equipped with a LC-3AD solvent delivery system, a SIL-30ACXR auto-sampler, a CTO-30AC column oven, a DGU-20A3 degasser and a CBM-20A controller. Chromatographic separation was conducted on a ACQUITY UPLC^®^BEH C_18_ (100 × 2.1 mm, 1.7 μm) keeping at 35 °C. 0.1% aqueous formic acid (v/v, A) and acetonitrile (B) were used as the mobile phase. The gradient elution with the flow rate of 0.3 mL/min was performed as follows: 0–5 min 5–15% B; 5–15 min 15–18% B; 15–25 min 18–35% B; 25–35 min 35–95% B; 35–37 min 95–95% B; 37–37.1 min 95–5% B; 37.1–40.0 min 5–5% B. The sample inject volume was 3 μL.

UHPLC-Q-TOF-MS/MS detection was conducted on a Triple TOF^TM^ 5600+ system with a Duo Spray source in both positive and negative electrospray ion mode (AB SCIEX, Foster City, CA, USA). The MS analysis was carried out by the ESI source in both positive- and negative-ion modes. The parameters were set as follows: ion spray voltage, −5500 V; ion source temperature, 500 °C; curtain gas, 40 psi; nebulizer gas (GS1), 50 psi; heater gas (GS2), 50 psi; and decluster potential (DP), -100 V. Mass ranges were set at 100–1500 Da for the TOF-MS scan and 100–1500 Da for the TOF MS/MS experiments. In the IDA-MS/MS experiment, the collision energy (CE) was set at 45 eV, and the collision energy spread (CES) was (±) 15 eV for the UHPLC-Q-TOF-MS/MS detection. The most intensive five ions from each TOF-MS scan were selected as MS/MS fragmentation. LC-MS/MS data were analyzed using PeakView^®^1.2 software (AB SCIEX, Foster City, CA, USA).

## 4. Conclusions

In this study, a rapid, efficient, and precise UHPLC-Q-TOF-MS/MS approach was developed for the separation and identification of the main compositions in the roots of *S. cathayensis* for the first time. By the virtue of high resolution and high separation speed of UHPLC, and accurate MS data of Q-TOF-MS, a total of 85 components, including 35 alkaloids, 12 flavonoids, 7 terpenoids, 5 phenylpropanoids, 9 fatty acids, 7 cyclic peptides, and 10 others were identified by comparisons of their retention times, accurate masses, fragment ions, related literatures. Moreover, alkaloid, flavonoid, phenylpropanoid, and cyclic peptide were reported from *S. cathayensis* for the first time. This rapid and sensitive method was highly useful to comprehend the chemical compositions and will provide a scientific basis for further study on the material basis, mechanism and clinical application of *S. cathayensis* roots.

## Figures and Tables

**Table 1:** Compounds identified from the roots of *S. cathayensis* by UHPLC–Q-TOF-MS/MS in positive ion mode.

No.	*t*_R_ (min)	Compounds	Molecular Formula	Molecular Weight	Measured Mass [M + H]	Error (ppm)	MS^2^	Ref.
**Alkaloids**								
4	1.74	stachydrine	C_7_H_13_NO_2_	143.0946	144.1020	0.8	144.1004 ^a^, 128.0647, 102.0585	[21]
6	4.06	gentianin	C_10_H_9_NO_2_	175.0633	176.0706	−0.2	176.0714 ^a^, 148.0763, 133.0524, 130.0660, 103.0560, 120.0820, 117.0350	[22]
7	4.10	kalacolidine	C_22_H_35_NO_5_	393.2515	394.2593	1.4	394.2588, 376.2485 ^a^	[23]
10	5.49	mesaconine	C_24_H_39_NO_9_	485.2625	486.2702	1.0	486.2697 ^a^, 436.2332, 404.2070	[23]
11	5.54	16-β-hydroxycardiopetaline	C_21_H_33_NO_4_	363.2410	364.2486	1.0	364.2488 ^a^, 346.2382, 328.2275	[24]
13	5.84	senbusine A	C_23_H_37_NO_6_	423.2621	424.2699	1.3	424.2701 ^a^, 406.2591, 388.2487	[23]
16	6.05	carmichaeline	C_22_H_35_NO_4_	377.2566	378.2645	1.6	378.2640, 360.2532 ^a^, 328.2271	[23]
18	6.10	isotalatizidine	C_23_H_37_NO_5_	407.2672	408.2749	1.2	390.2642 ^a^, 358.2378	[23]
19	6.46	aconine	C_25_H_41_NO_9_	499.2781	500.2861	1.3	500.2863 ^a^, 450.2495	[15]
20	6.56	songorine	C_22_H_31_NO_3_	357.2304	358.2381	1.2	358.2375, 340.2270 ^a^	[23]
21	6.6	napelline	C_22_H_33_NO_3_	359.2460	360.2536	0.7	360.2536, 342.2430 ^a^	[15]
22	6.74	hetisine	C_20_H_27_NO_3_	329.1991	330.2069	1.6	330.2062 ^a^, 312.1954	[23]
25	7.38	hypaconine	C_24_H_39_NO_8_	469.2676	470.2751	0.6	470.2764 ^a^, 438.2498, 406.2216	[24]
26	7.44	senbusine C	C_24_H_39_NO_7_	453.2727	454.2805	1.3	454.2786 ^a^, 404.2427	[23]
28	7.91	neoline	C_24_H_39_NO_6_	437.2777	438.2854	0.8	438.2841 ^a^, 420.2738, 388.2480, 356.2222, 154.1223	[23]
29	8.08	14-acetyl-karakoline	C_24_H_37_NO_5_	419.2672	420.2749	1.0	420.2757 ^a^, 402.2653	[24]
35	9.18	talatisamine	C_24_H_39_NO_5_	421.2828	422.2906	1.2	390.2642 ^a^, 358.2379	[24]
37	9.41	denudatine	C_22_H_33_NO_2_	343.2511	344.2589	1.4	344.2587 ^a^, 326.2480	[23]
42	10.76	bullatine C	C_26_H_41_NO_7_	479.2883	480.2962	1.2	480.2980 ^a^, 462.2858, 430.2587, 398.2295	[23]
43	11.22	chasmanine	C_25_H_41_NO_6_	451.2934	452.3012	1.2	452.3016 ^a^, 420.2755, 388.2490	[23]
44	13.41	14-acetyl-talatisamine	C_26_H_41_NO_6_	463.2934	464.3011	1.0	464.3011 ^a^, 432.2746	[23]
50	20.28	benzoylmesaconine	C_31_H_43_NO_10_	589.2887	590.2966	1.1	590.2941 ^a^, 540.2579, 508.2323, 105.0341	[15]
54	21.85	benzoylaconine	C_32_H_45_NO_10_	603.3044	604.3122	0.9	604.3110 ^a^, 554.2748	[15]
55	22.91	benzoylhypaconine	C_31_H_43_NO_9_	573.2938	574.3015	0.8	574.3017 ^a^, 542.2755, 510.2495	[15]
58	24.20	benzoyl-3,13-deoxymesaconine	C_31_H_43_NO_8_	557.2989	558.3068	1.1	558.3055 ^a^, 540.2955, 508.2697	[15]
59	24.25	10-hydroxy-mesaconitine	C_33_H_45_NO_12_	647.2942	648.3024	1.4	648.3002 ^a^, 588.2791, 556.2540, 370.1653	[23]
60	24.27	benzoyldeoxyaconine	C_32_H_45_NO_9_	587.3094	588.3172	0.8	588.3174 ^a^, 556.2918	[15]
61	25.21	pyrohypaconitine	C_31_H_41_NO_8_	555.2832	556.2910	0.9	556.2915 ^a^, 524.2647, 492.2414, 452.2072, 402.2285, 238.1807, 192.1383	[15]
63	25.87	mesaconitine	C_33_H_45_NO_11_	631.2993	632.3070	0.7	632.3032 ^a^, 572.2826, 540.2566, 512.2622, 508.2315, 354.1685, 105.0342	[15]
64	26.12	10-hydroxy-aconitine	C_34_H_47_NO_12_	661.3098	662.3180	1.4	662.3168 ^a^, 602.2957, 570.2702, 384.1809	[15]
67	27.49	hypaconitine	C_33_H_45_NO_10_	615.3044	616.3125	1.5	616.3089 ^a^, 556.2876, 524.2618, 496.2638, 342.2055, 338.1739, 105.0340	[15]
68	27.58	aconitine	C_34_H_47_NO_11_	645.3149	646.3232	1.5	646.3199 ^a^, 586.2985, 554.2735, 526.2783, 522.2487, 368.1850, 105.0342	[23]
70	28.60	deoxyaconitine	C_34_H_47_NO_10_	629.3200	630.3281	1.3	630.3256 ^a^, 570.3047, 538.2787, 510.2830, 478.2575, 356.2219, 352.1905	[15]
71	28.98	yunaconitine	C_35_H_49_NO_11_	659.3306	660.3386	1.2	660.3396 ^a^, 572.2866, 540.2591, 354.1735	[24]
72	29.38	3,13-dideoxyaconitine	C_34_H_47_NO_9_	613.3251	614.3326	0.4	614.3302 ^a^, 554.3091, 522.2835, 494.2880, 462.2620, 105.0345	[23]
**Terpenoids**								
14	5.94	oxypaeoniflorin	C_23_H_28_O_12_	496.1581	497.1657	0.7	497.2676, 349.1575, 197.0831, 133.0687, 121.0297 ^a^	[25]
31	8.56	paeoniflorin	C_23_H_28_O_11_	480.1632	481.1711	1.4	319.1245, 197.0808, 179.0691,151.0750, 133.0650, 105.0342 ^a^	[26]
65	26.21	benzoylpaeoniflorin	C_30_H_32_O_12_	584.1894	585.1970	0.5	585.3271, 319.1195, 267.0885, 249.0785, 197.0807, 179.0705, 121.0666,105.0349 ^a^	[26]
73	30.98	atractylenolide-1	C_15_H_18_O_2_	230.1307	231.1380	0.0	213.1257, 163.0778, 155.0848, 143.0931, 128.0610, 115.0541, 105.0712 ^a^	[27]
79	36.18	3-oxo-olean-12-en-28-oic acid	C_30_H_46_O_3_	454.3447	455.3516	−0.8	455.3539, 437.3426, 247.1668, 233.1531, 229.1584, 197.1332, 189.1615 ^a^	[28,29]

^a^ base peak.

**Table 2:** Compounds identified from the roots of *S. cathayensis* by UHPLC–Q-TOF-MS/MS in negative ion mode

No.	*t*_R_ (min)	Compounds	Molecular Formula	Molecular Weight	Measured Mass [M − H]^−^	Error (ppm)	MS^2^	Ref.
**Flavonoids**								
24	7.33	puerarin	C_21_H_20_O_9_	416.1107	415.1015	−4.6	415.0970, 295.0614, 277.0490, 267.0653 ^a^	[30]
33	8.85	scoparin	C_22_H_22_O_11_	462.1162	461.1069	−4.4	415.0991 ^a^, 252.0361	[30]
34	9.17	isorhamnetin-3-*O*-neohespeidoside	C_28_H_32_O_16_	624.1690	623.1602	−2.5	623.1609, 461.1022 ^a^, 417.1203, 315.0710, 153.0226, 145.0338	[31]
36	9.38	kaempferol-3-*O*-glucorhamnoside	C_27_H_30_O_15_	594.1585	593.1485	−4.6	593.1010, 547.1243, 430.0954, 275.0347,112.9889 ^a^	[32]
39	9.44	methyl hesperidin	C_29_H_36_O_15_	624.2054	623.1963	−2.9	623.0455, 577.0923, 534.0988, 461.1451, 410.0366, 315.1067, 145.0319 ^a^	[33]
17	6.06	catechin	C_15_H_14_O_6_	290.0790	289.0715	−1.1	221.0899, 205.0532, 203.0700 ^a^, 187.0372, 159.0452, 125.0280, 123.0486	[34]
46	17.01	naringin	C_27_H_32_O_14_	580.1792	579.1716	−0.6	579.1721, 459.1144, 313.0736, 271.0617 ^a^, 177.0209, 151.0048	[33]
48	19.58	hesperidin	C_28_H_34_O_15_	610.1898	609.1815	−1.6	609.1826, 301.0706 ^a^, 286.0481, 242.0583	[35]
56	23.57	5,8-dihydroxy-6,7-dimethoxyflavone	C_17_H_14_O_6_	314.0790	313.0709	−2.9	297.0313, 283.0226, 266.0197, 255.0309 ^a^, 227.0318, 211.0393, 185.0235, 183.0456	[33]
57	24.10	juglanin	C_20_H_18_O_10_	418.0900	417.0814	−3.1	161.0578, 135.0527, 129.0226 ^a^	[20]
62	25.32	naringenin	C_15_H_12_O_5_	272.0685	271.0609	−1.1	151.0030, 119.0509 ^a^, 117.0421	[33]
66	26.53	hesperetin	C_16_H_14_O_6_	302.0790	301.0713	−1.7	258.0578 ^a^, 134.0383	[33]
**Terpenoids**								
76	33.74	2α,3β-dihydroxyolean-12-en-28-oic-acid	C_30_H_48_O_4_	472.3553	471.3466	−3.0	471.3494 ^a^, 451.0162, 411.0302, 389.2158, 330.9965, 264.9917	[28,29]
81	36.45	oleanic acid	C_30_H_48_O_3_	456.3604	455.3512	−4.2	455.3495 ^a^, 409.2443, 391.2341, 373.2227, 355.2079	[28,29]
**Phenylpropanoids**								
15	6.01	ferulic acid	C_10_H_10_O_4_	194.0579	193.0510	2.0	134.0379 ^a^	[36]
22	7.10	fraxin	C_16_H_18_O_10_	370.0900	369.0814	−3.5	223.0462 ^a^, 205.0350, 129.0210, 125.0241	[32]
32	8.76	3-(3,4-dihydroxyphenyl)-2-hydroxy-propanoic acid	C_9_H_10_O_5_	198.0528	197.0456	0.2	162.8375 ^a^, 160.8401, 138.0358, 123.0085	[37]
38	9.44	acteoside	C_29_H_36_O_15_	624.2054	623.1963	−2.9	623.0455, 577.0923, 461.1451, 315.1067, 145.0319 ^a^	[30]
52	21.42	bergaptol	C_11_H_6_O_4_	202.0266	201.0192	−0.8	228.9172 ^a^, 166.8855, 147.8874, 117.0436	[38]
**Fatty Acids**								
5	2.05	citric acid	C_6_H_8_O_7_	192.0270	191.0199	0.7	146.9074, 111.0110 ^a^	[36]
69	28.24	trihydroxy-octadecaenoic acid	C_18_H_34_O_5_	330.2406	329.2326	−1.5	329.2354, 229.1443, 211.1346, 183.1371, 171.1026 ^a^, 139.1137	[27]
74	32.9	dihydroxy-octadecatrienoic acid	C_18_H_30_O_4_	310.2144	309.2069	−0.9	291.1995 ^a^, 199.8548, 179.1442, 110.0373	[27]
77	34.13	hydroxy-octadecatrienoic acid	C_18_H_30_O_3_	294.2195	293.2121	−0.4	293.2080 ^a^, 199.8526, 149.0939, 125.1018	[28]
78	36.16	linolenic acid	C_18_H_30_O_2_	278.2246	277.2171	−0.7	134.8951 ^a^	[28]
80	36.22	stearic acid	C_18_H_36_O_2_	284.2715	283.2640	−0.9	283.2633, 199.8512 ^a^	[28]
83	37.05	linoleic acid	C_18_H_32_O_2_	280.2402	279.2329	−0.2	279.2319 ^a^, 261.2194	[28]
84	38.09	palmitic acid	C_16_H_32_O_2_	256.2402	255.2332	0.9	256.2333, 255.2327 ^a^, 114.9333	[28]
85	38.20	oleic acid	C_18_H_34_O_2_	282.2559	281.2484	−0.9	281.2489 ^a^	[28]
**Cyclic Peptides**								
27	7.59	cyclo trileucyl (or isoleucyl)	C_18_H_33_N_3_O_3_	339.2522	384.2488 ^b^	−1.3	135.0456 ^a^	[28]
40	10.07	cyclo tetraleucyl (or isoleucyl)	C_24_H_44_N_4_O_4_	452.3363	497.3328 ^b^	−1.1	497.1555, 451.3294 ^a^, 433.3159, 337.2669, 224.1758, 137.0247	[28]
45	13.48	cyclo pentaleucyl (or isoleucyl)	C_30_H_55_N_5_O_5_	565.4203	610.4168 ^b^	−1.0	564.4112 ^a^, 546.4021, 225.1592	[28]
47	17.66	cyclo hexaleucyl (or isoleucyl)	C_36_H_66_N_6_O_6_	678.5044	723.5020 ^b^	0.7	677.4961 ^a^	[28]
49	19.86	cyclo hetaleucyl (or isoleucyl)	C_42_H_77_N_7_O_7_	791.5885	836.5862 ^b^	0.8	790.5791 ^a^	[28]
51	21.0	cyclo octaleucyl (or isoleucyl)	C_48_H_88_N_8_O_8_	904.6725	949.6704 ^b^	0.9	946.6691, 903.6636 ^a^	[28]
53	21.78	cyclo nonaleucyl (or isoleucyl)	C_54_H_99_N_9_O_9_	1017.7566	1062.7546 ^b^	0.9	1062.7547, 1016.7472 ^a^	[28]
**Others**								
1	0.35	glucogallin	C_13_H_16_O_10_	332.0744	331.0662	−2.7	169.0124, 125.0257 ^a^	[39]
2	0.69	gallic acid	C_7_H_6_O_5_	170.0215	169.0149	3.6	124.0178 ^a^	[40]
3	1.46	sucrose	C_12_H_22_O_11_	342.1162	341.1078	−3.5	221.0641, 179.0592, 161.0419, 119.0379, 113.0253 ^a^	[41]
8	4.29	piscidic acid	C_11_H_12_O_7_	256.0583	255.0508	−0.7	218.8641, 180.9830, 165.0550 ^a^, 118.9815	[42]
9	5.37	vanillin	C_8_H_8_O_3_	152.0473	151.0410	4.1	105.0368 ^a^	[43]
12	5.59	protocatechuic acid pentoside	C_12_H_14_O_8_	286.0689	285.0611	−1.6	152.0117, 108.0243 ^a^	[44]
30	8.37	vanillic acid	C_8_H_8_O_4_	168.0423	167.0354	2.2	108.0206 ^a^	[36]
41	10.65	paeonol	C_9_H_10_O_3_	166.0630	165.0564	3.8	147.0476, 119.0505, 117.0379 ^a^, 103.0575, 101.0392	[31]
75	33.10	dibutyl phthalate	C_16_H_22_O_4_	278.1518	277.1444	−0.3	147.0072 ^a^	[45]
82	36.52	dimethisterone	C_23_H_32_O_2_	340.2402	339.2326	−1.1	339.2317, 163.1140 ^a^	[45]

^a^ base peak, ^b^ measured mass [M + HCOO]^−^.

## Supplementary Materials

The following are available online, Figure S1: The tandem mass spectra of compound 25 (hypaconine) in positive ion mode, Figure S2: The tandem mass spectra of compound 26 (senbusine C) in positive ion mode, Figure S3: The tandem mass spectra of compound 28 (neoline) in positive ion mode, Figure S4: The tandem mass spectra of compound 50 (benzoylmesaconine) in positive ion mode, Figure S5: The tandem mass spectra of compound 58 (benzoyl-3,13-deoxymesaconine) in positive ion mode, Figure S6: The tandem mass spectra of compound 59 (10-hydroxy-mesaconitine) in positive ion mode, Figure S7: The tandem mass spectra of compound 60 (benzoyldeoxyaconine) in positive ion mode, Figure S8: The tandem mass spectra of compound 63 (mesaconitine) in positive ion mode, Figure S9: The tandem mass spectra of compound 64 (10-hydroxy-aconitine) in positive ion mode, Figure S10: The tandem mass spectra of compound 67 (hypaconitine) in positive ion mode, Figure S11: The tandem mass spectra of compound 68 (aconitine) in positive ion mode, Figure S12: The tandem mass spectra of compound 70 (deoxyaconitine) in positive ion mode, Figure S13: The tandem mass spectra of compound 72 (3,13-dideoxyaconitine) in positive ion mode, Figure S14: The tandem mass spectra of compound 36 (kaempferol-3-*O*-glucorhamnoside) in negative ion mode, Figure S15: The tandem mass spectra of compound 46 (naringin) in negative ion mode, Figure S16: The tandem mass spectra of compound 48 (hesperidin) in negative ion mode, Figure S17: The tandem mass spectra of compound 38 (acteoside) in negative ion mode, Figure S18: The tandem mass spectra of compound 83 (linoleic acid) in negative ion mode.
