# Supplementary material for: Rapid Analysis of the Chemical Compositions in Semiliquidambar cathayensis Roots by Ultra High-Performance Liquid Chromatography and Quadrupole Time-of-Flight Tandem Mass Spectrometry
Source: Molecules. 2019 Nov 13;24(22):4098. doi: 10.3390/molecules24224098 (PMC6891699; doi:10.3390/molecules24224098)
Supplement: Supplementary file 1 [file molecules-24-04098-s001.pdf]

Supplementary Data

Rapid Analysis of the Chemical Compositions in *Semiliquidambar cathayensis* Roots by Ultra high-performance Liquid Chromatography and Quadrupole Time-of-flight Tandem Mass Spectrometry

Li Yang [1], Rong-Hua Liu [2,*] and Jun-Wei He [3,*]

[1] Key Laboratory of Modern Preparation of TCM, Jiangxi University of Traditional Chinese Medicine, Ministry of Education, Nanchang 330004, China; yangli07971@163.com (L.Y.)

[2] College of Pharmacy, Jiangxi University of Traditional Chinese Medicine, Nanchang 330004, China; rhliu@163.com (R.-H.L)

[3] Research Center of Natural Resources of Chinese Medicinal Materials and Ethnic Medicine,
Jiangxi University of Traditional Chinese Medicine, Nanchang 330004, China; hjwjn2008@163.com (J.-W.H.)

* Correspondence: hjwjn2008@163.com (J.-W.H); Tel.: +86-791-87118873 (J.-W.H); rhliu@163.com (R.-H.L).

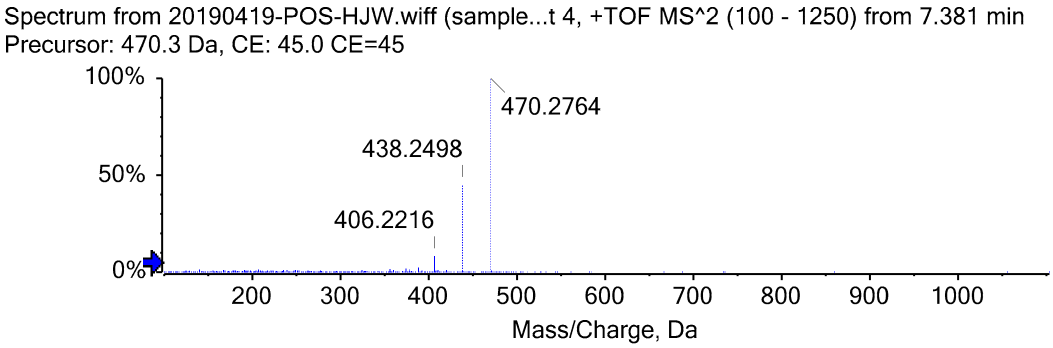

Figure S1 The tandem mass spectra of compound **25** (hypaconine) in positive ion mode

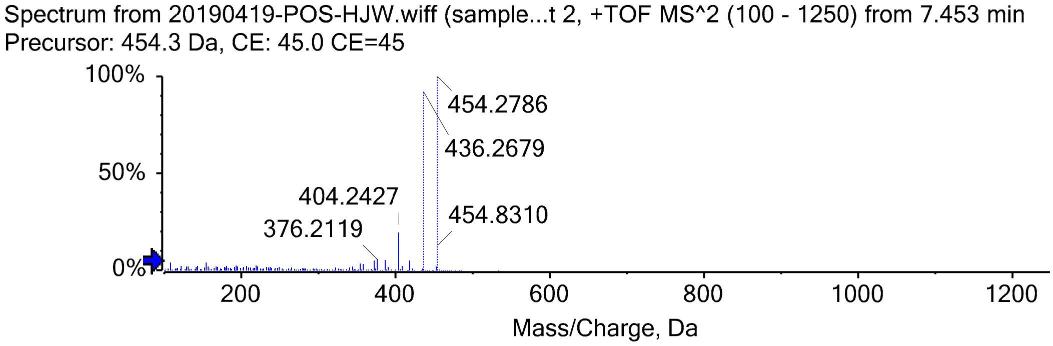

Figure S2 The tandem mass spectra of compound **26** (senbusine C) in positive ion mode.

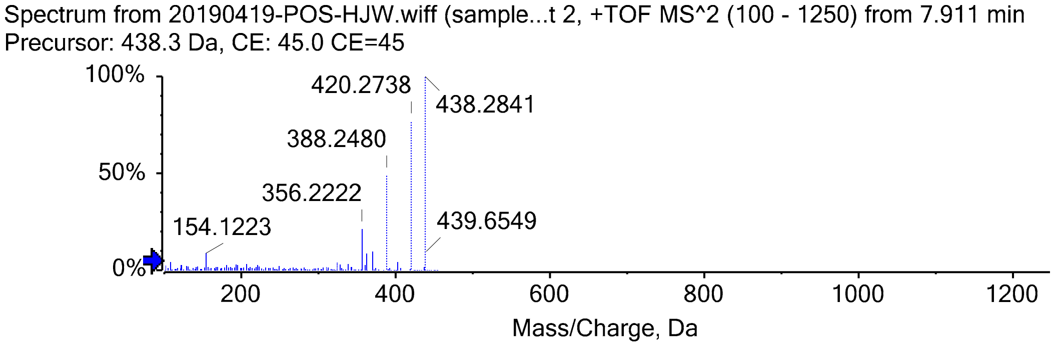

Figure S3 The tandem mass spectra of compound **28** (neoline) in positive ion mode.

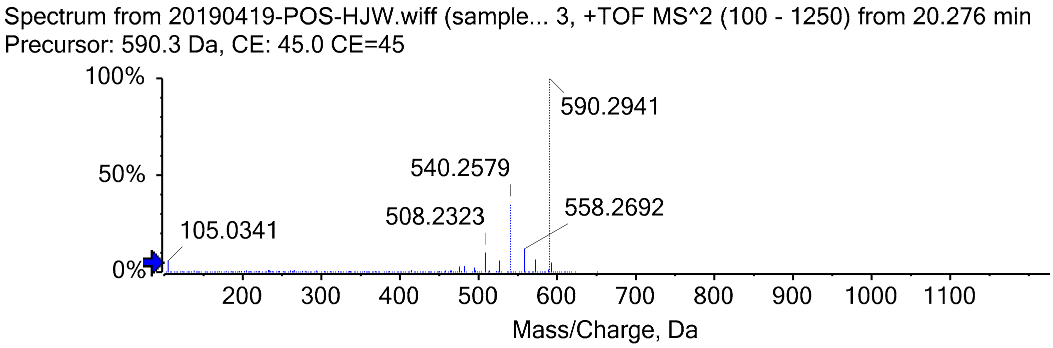

Figure S4 The tandem mass spectra of compound **50** (benzoylmesaconine) in positive ion mode.

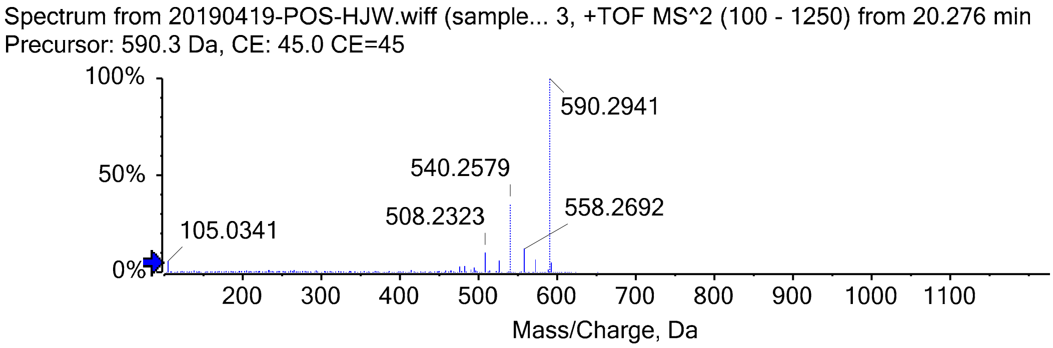

Figure S5 The tandem mass spectra of compound **58** (benzoyl-3,13-deoxymesaconine) in positive ion mode.

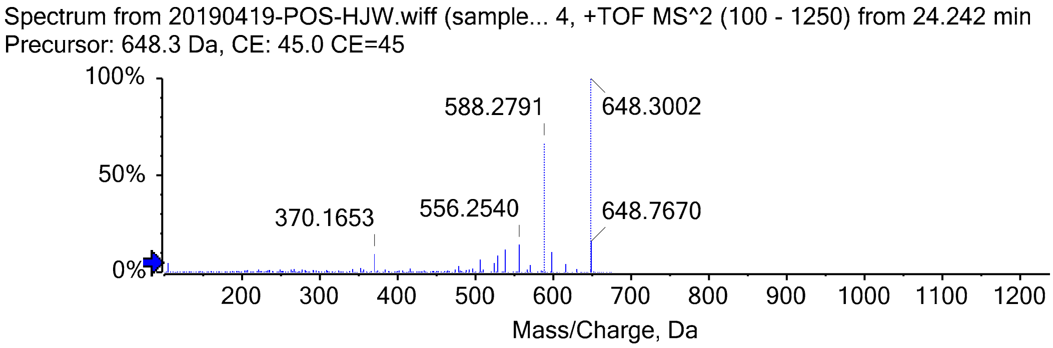

Figure S6 The tandem mass spectra of compound **59** (10-hydroxy-mesaconitine) in positive ion mode.

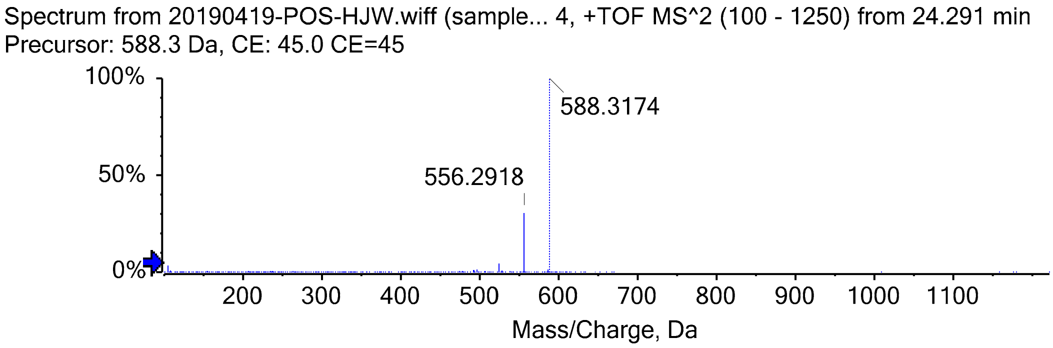

Figure S7 The tandem mass spectra of compound **60** (benzoyldeoxyaconine) in positive ion mode.

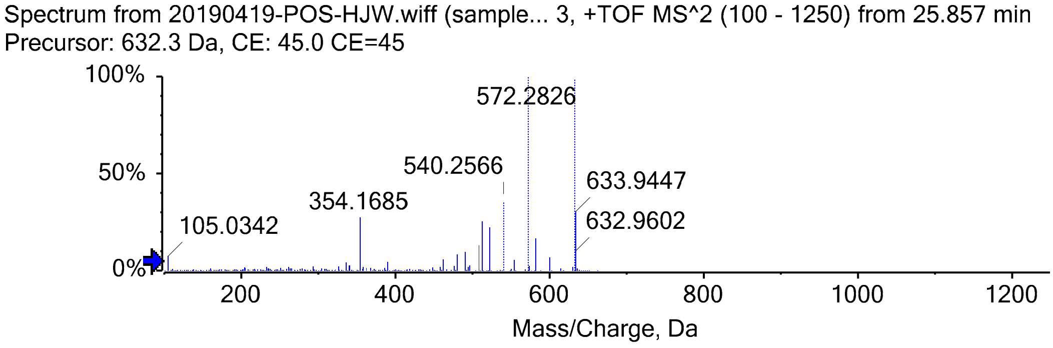

Figure S8 The tandem mass spectra of compound **63** (mesaconitine) in positive ion mode.

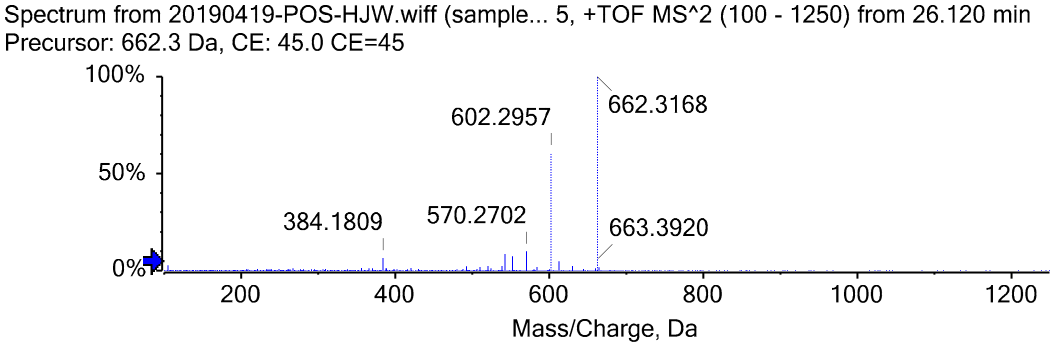

Figure S9 The tandem mass spectra of compound **64** (10-hydroxy-aconitine) in positive ion mode.

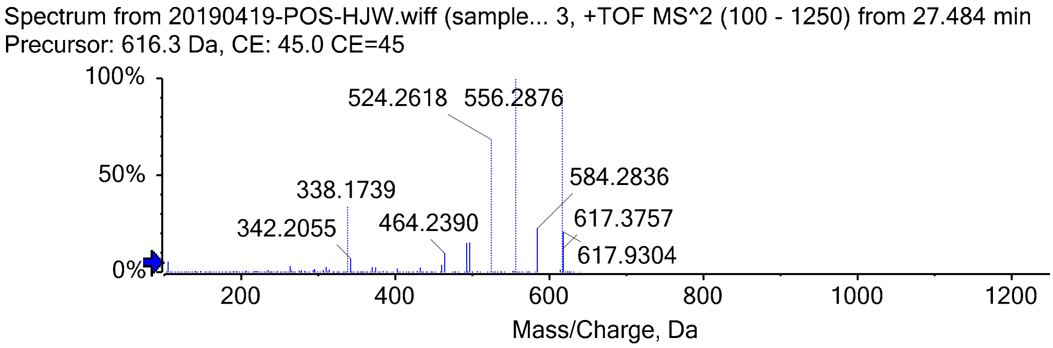

Figure S10 The tandem mass spectra of compound **67** (hypaconitine) in positive ion mode.

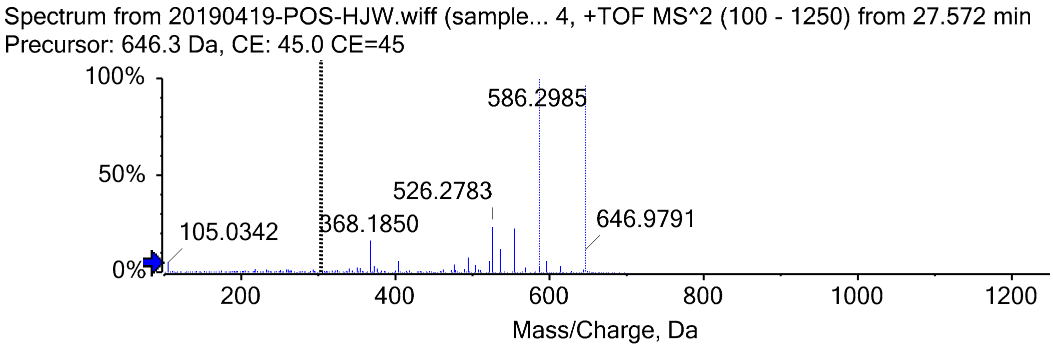

Figure S11 The tandem mass spectra of compound **68** (aconitine) in positive ion mode.

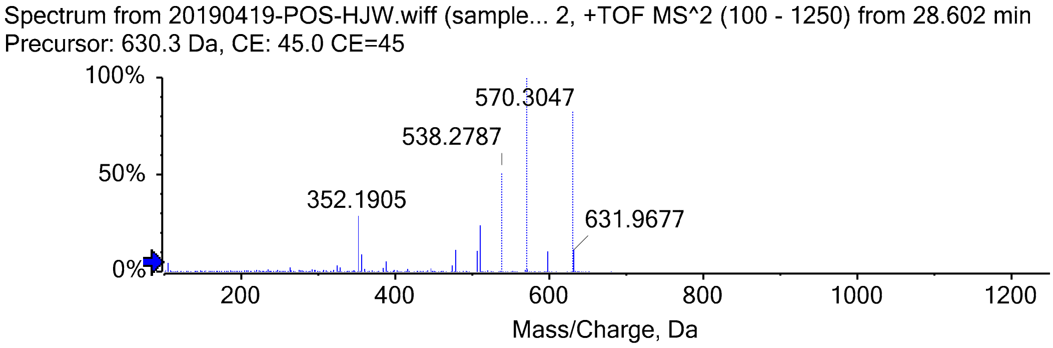

Figure S12 The tandem mass spectra of compound **70** (deoxyaconitine) in positive ion mode.

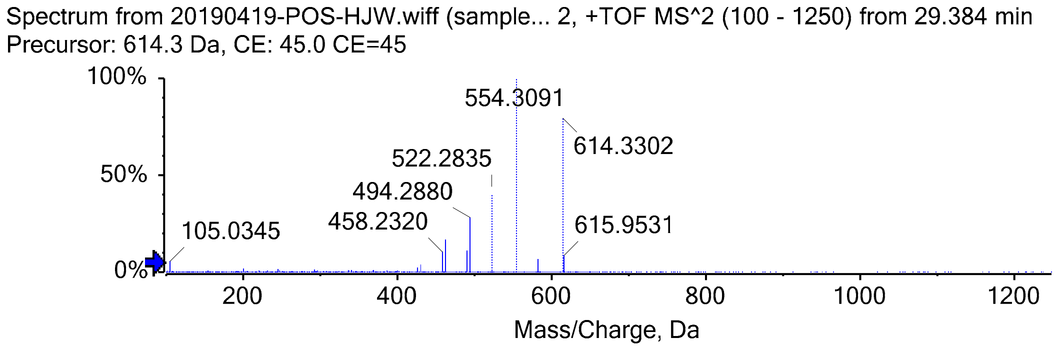

Figure S13 The tandem mass spectra of compound **72** (3,13-dideoxyaconitine) in positive ion mode.

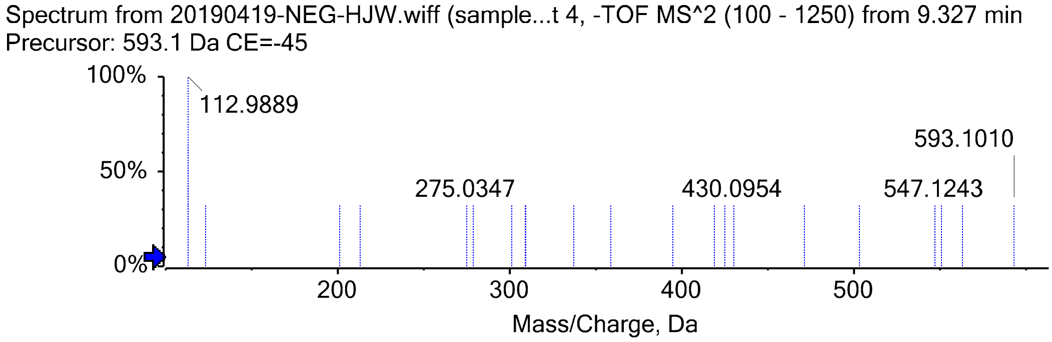

Figure S14 The tandem mass spectra of compound **36** (kaempferol-3-*O*-glucorhamnoside) in negative ion mode.

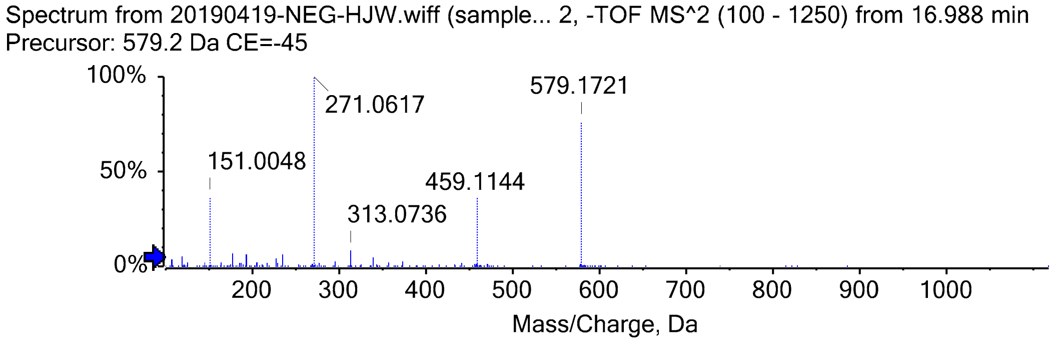

Figure S15 The tandem mass spectra of compound **46** (naringin) in negative ion mode.

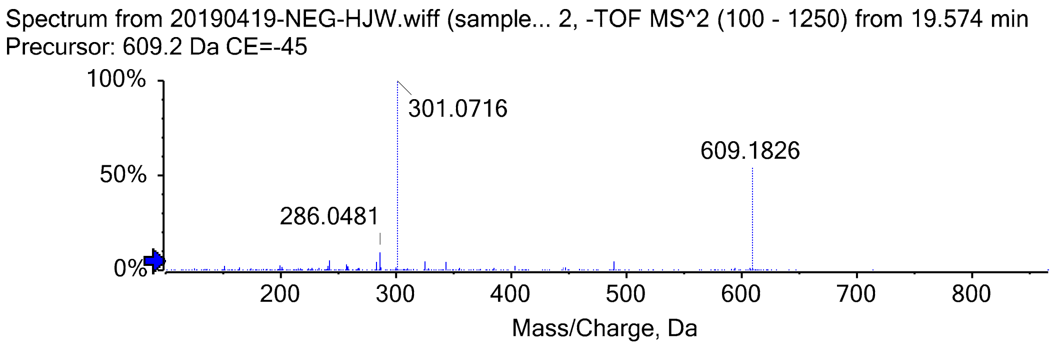

Figure S16 The tandem mass spectra of compound **48** (hesperidin) in negative ion mode.

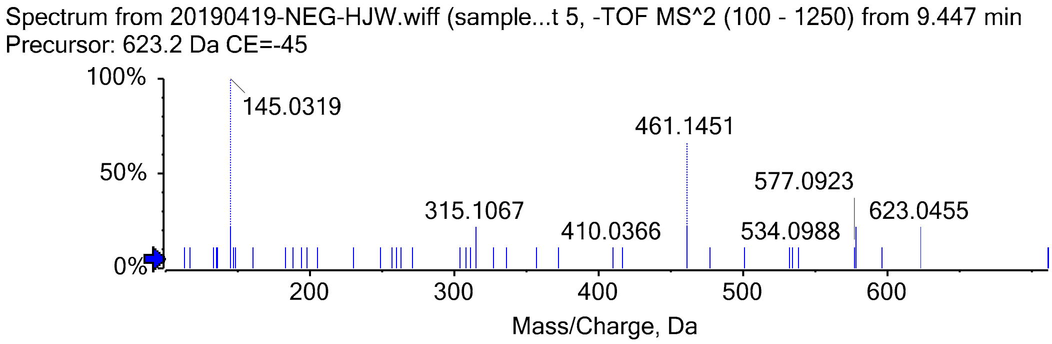

Figure S17 The tandem mass spectra of compound **38** (acteoside) in negative ion mode.

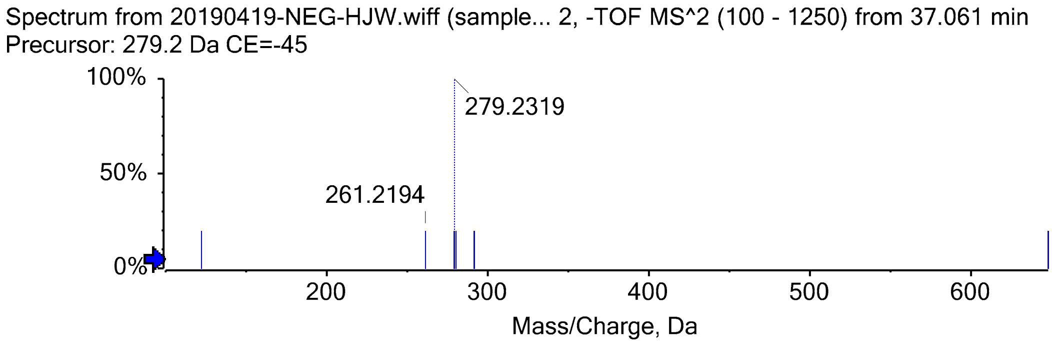

Figure S18 The tandem mass spectra of compound **83** (linoleic acid) in negative ion mode.
